# Child Mortality after Discharge from a Health Facility following Suspected Pneumonia, Meningitis or Septicaemia in Rural Gambia: A Cohort Study

**DOI:** 10.1371/journal.pone.0137095

**Published:** 2015-09-09

**Authors:** Aakash Varun Chhibber, Philip C. Hill, James Jafali, Momodou Jasseh, Mohammad Ilias Hossain, Malick Ndiaye, Jayani C. Pathirana, Brian Greenwood, Grant A. Mackenzie

**Affiliations:** 1 Centre for International Health, University of Otago, Dunedin, New Zealand; 2 Medical Research Council (UK), The Gambia Unit, Fajara, The Gambia; 3 Faculty of Infectious and Tropical Diseases, London School of Hygiene & Tropical Medicine, London, United Kingdom; 4 Infection & Immunity Theme, Murdoch Childrens Research Institute, Melbourne, Australia; University British Columbia, CANADA

## Abstract

**Objective:**

To measure mortality and its risk factors among children discharged from a health centre in rural Gambia.

**Methods:**

We conducted a cohort study between 12 May 2008 and 11 May 2012. Children aged 2–59 months, admitted with suspected pneumonia, sepsis, or meningitis after presenting to primary and secondary care facilities, were followed for 180 days after discharge. We developed models associating post-discharge mortality with clinical syndrome on admission and clinical risk factors.

**Findings:**

One hundred and five of 3755 (2.8%) children died, 80% within 3 months of discharge. Among children aged 2–11 and 12–59 months, there were 30 and 29 deaths per 1000 children per 180 days respectively, compared to 11 and 5 respectively in the resident population. Children with suspected pneumonia unaccompanied by clinically severe malnutrition (CSM) had the lowest risk of post-discharge mortality. Mortality increased in children with suspected meningitis or septicaemia without CSM (hazard ratio [HR] 2.6 and 2.2 respectively). The risk of mortality greatly increased with CSM on admission: CSM with suspected pneumonia (HR 8.1; 95% confidence interval (CI) 4.4 to 15), suspected sepsis (HR 18.4; 95% CI 11.3 to 30), or suspected meningitis (HR 13.7; 95% CI 4.2 to 45). Independent associations with mortality were: mid-upper arm circumference (MUAC) of 11.5–13.0 cm compared to >13.0 cm (HR 7.2; 95% CI 3.0 to 17.0), MUAC 10.5–11.4 cm (HR 24; 95% CI 9.4 to 62), and MUAC <10.5 cm (HR 44; 95% CI 18 to 108), neck stiffness (HR 10.4; 95% CI 3.1 to 34.8), non-medical discharge (HR 4.7; 95% CI 2.0 to 10.9), dry season discharge (HR 2.0; 95% CI 1.2 to 3.3), while greater haemoglobin (HR 0.82; 0.73 to 0.91), axillary temperature (HR 0.71; 95% CI 0.58 to 0.87), and oxygen saturation (HR 0.96; 95% CI 0.93 to 0.99) were associated with reduced mortality.

**Conclusion:**

Gambian children experience increased mortality after discharge from primary and secondary care. Interventions should target both moderately and severely malnourished children.

## Background

Millennium Development Goal 4, reduction in under-5 mortality by two-thirds by 2015, is unlikely to be met in many countries [1, 2]. The highest rates of child mortality are in sub-Saharan Africa [3]. Infectious diseases are responsible for nearly two-thirds of these deaths and under-nutrition is a contributing factor in at least a third [4].

The WHO program for Integrated Management of Childhood Illness focuses on rational treatments for syndromic presentations at first-level health facilities and also provides advice on follow-up care [5]. In sub-Saharan Africa, there is little focus on follow-up, because of under-resourced health systems with many competing priorities. Additional data on post-discharge mortality in settings of primary and secondary care could help decisions about the level of priority given to follow-up care.

Child mortality after discharge from hospital in high-income countries is confined to small high risk groups [6–8]. In low-income settings, children appear to be at increased risk of mortality following any illness [9–13]. Estimates of post-discharge mortality have varied between 1% and 18% often with a similar or greater magnitude than in-hospital mortality [13]. Mortality following specific illnesses varies, as do the results of risk factor analyses [9–12, 14, 15]. The most important factors associated with post-discharge mortality appear to be young age, malnutrition, previous hospitalisations, HIV infection, and pneumonia [13]. Most studies of post-discharge mortality in low-income settings have involved referral centres [9, 10, 12, 14, 15]. To obtain data more representative of the majority of admissions in sub-Saharan Africa we determined the risk factors for and rates of child mortality following discharge of children in a setting of primary and secondary care within a demographic surveillance system in rural West Africa.

## Methods

### Setting

We conducted a prospective cohort study of children discharged from Basse Health Centre in eastern Gambia. *Plasmodium falciparum* is endemic in the region with seasonal transmission during the brief wet season from June until October. The region is rural and the population engages primarily in subsistence farming. Basse is the major town in Upper River Region (URR). The health centre has 12 paediatric beds, providing primary and secondary care to referrals from five peripheral nurse-led clinics located on the south bank of the River Gambia. There are no private inpatient facilities in the area. The Basse Health and Demographic Surveillance System (BHDSS) comprises the geographic area of URR to the south of the river (S1 Fig).

Demographic surveillance includes 4-monthly visits to all households during which births, deaths, and migrations are registered. Residency is defined as living in the BHDSS area for two consecutive visits or being born to a woman resident in the area. Loss of residency is defined as absence for two consecutive visits. In 2010, the population was estimated to be 160,634 with under-5 mortality of 81 per 1000 live births. Surveillance for cases of suspected pneumonia, sepsis, and meningitis in the area has been underway since 2008 and all cases are linked to a unique individual BHDSS identity number.

### Participants

Children were eligible for inclusion in the cohort if they were admitted to Basse Health Centre between 12 May 2008 and 30 April 2012, a BHDSS resident, 2–59 months of age, met criteria for suspected pneumonia, meningitis or septicaemia [16], and were discharged alive. The proportion of all children admitted between 1 May 2011 and 30 April 2012 who met criteria for suspected pneumonia, meningitis or septicaemia was 77.9% (2224/2854). Children were ineligible if they were non-resident, discharged on the same date as admission or if they died on the day of discharge. If an individual was readmitted and discharged within the first 180 day post-discharge follow-up period, they contributed person-time for both admissions. Children contributed person-time to follow-up until one of the following occurred: exit from the BHDSS, attained 5 years of age, death, completed 180 day follow-up, or the study end (1 May 2012), which ever occurred first.

### Procedures

All individuals who presented as outpatients or inpatients to the six health facilities in the BHDSS were assessed by surveillance nurses 24 hours a day, 7 days a week, to determine if surveillance criteria for review by a clinician were present (S1 Table). We used a wide set of surveillance criteria for referral so as to include all patients who might meet diagnostic criteria for suspected pneumonia, sepsis, or meningitis. The referred patients did not necessarily require a higher level of clinical care. Clinicians used standardised criteria to make a surveillance diagnosis of suspected pneumonia, sepsis, or meningitis (S2 Table). Standardised investigations were performed on the basis of the surveillance diagnosis (S1 Panel). Patients were treated as per government guidelines.

The following clinical information was collected: presence of cough, difficulty breathing, irritability, convulsion, vomiting or diarrhea (three or more abnormally loose stool in the previous 24 hours), antibiotics taken in the previous week, the number of days of illness, axillary temperature, pulse rate, peripheral arterial oxygen saturation (Nellcor N-65, Mansfield, MA, USA), respiratory rate counted for 60 seconds (Respiratory Rate Counter, UNICEF, Copenhagen, Denmark), grunting, lower chest wall indrawing, nasal flaring, auscultatory crackles, wheeze, or bronchial breathing, dullness on chest percussion, bulging fontanel, neck stiffness, prostration (inability to drink or breastfeed or sit if usually able), conscious state (‘unresponsive’, ‘responds to pain’, ‘responds to voice’, or ‘alert’), and clinically severe malnutrition (CSM; visible wasting of the buttocks, skin or hair changes, or bilateral pedal edema). Haemoglobin concentration was measured systematically from January 2011 and at the clinician’s discretion during 2008–2010, using a point-of-care device (HemoCue Hb 301, Angelholm, Sweden), weight was recorded using a digital scale (HD-314, TANITA, Arlington Heights, Illinois, USA), height using a ShorrBoard (Weigh and Measure, Olney, USA), and mid-upper arm circumference (MUAC) using a tri-coloured single-slotted tape (Weigh and Measure, Olney, USA). Weight-for-height z-scores were calculated using 2006 WHO growth standards. A non-medical discharge was recorded if the patient left the health centre against medical advice. The wet season was defined as 1 July to 30 November and dry season as 1 December to 30 June. Deaths were monitored at 4-monthly household visits and we used the BHDSS dates of death. Inconsistent dates and child identities were resolved at additional household visits.

### Ethical considerations

The Gambia Government/MRC Joint Institutional Ethics Committee approved the project (SCC1087 and L2012.41). Surveillance nursing staff administered an informed and written consent process with the caregivers of each child enrolled in the study.

### Statistical analysis

Analysis was conducted using STATA version 12.0 (StataCorp. College Station, USA). Categorical variables were compared using Fishers exact or *χ* ^2^ tests. Variances of continuous variables were tested and the appropriate t-test was conducted to test differences between means. A survival analysis was used to model events within 180 days of discharge. Univariate Cox regression was used to identify associations with post-discharge mortality. Variables with *p* ≤0.10 on univariate analysis were entered into two multi-variable Cox regression models. A syndrome-based model used the standardised surveillance diagnoses of suspected pneumonia, sepsis, or meningitis in the presence or absence of CSM. Multiple syndromic diagnoses were categorised according to a mutually exclusive hierarchy of severity (meningitis > sepsis > pneumonia). A second model used individual clinical risk factors to determine associations with outcome. Variables with the highest *p*-value were removed from the multi-variable models one-by-one in a reverse stepwise approach until all *p*-values were <0.05.

## Results

**Fig 1:**
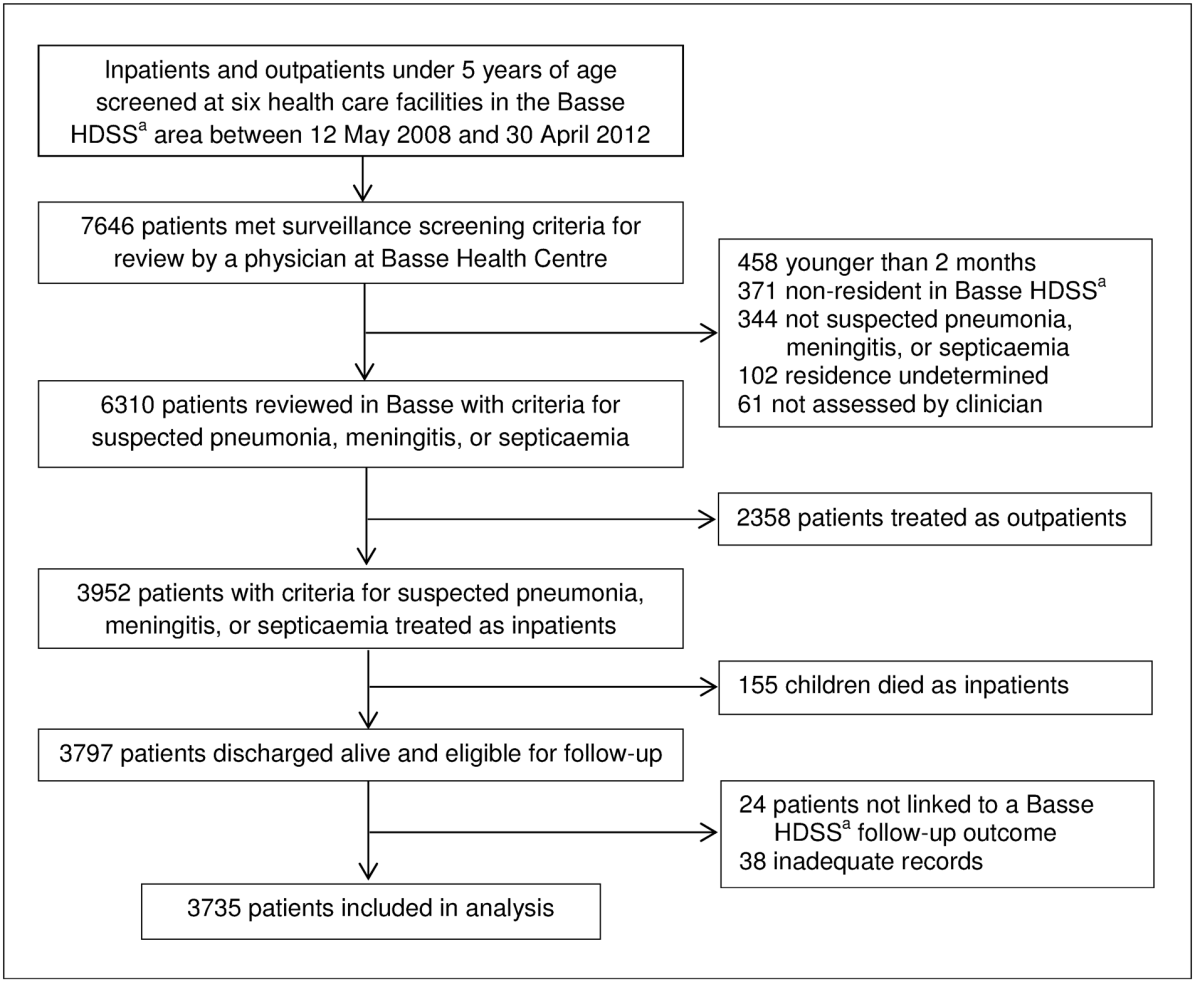
Flow diagram showing the study profile of patient enrolment, inclusion and exclusion, and loss-to-follow-up. This flowchart shows the numbers of participants who were screened, met criteria for enrolment, discharged alive and included in analysis. ^a^Basse Health and Demographic Surveillance System (Basse HDSS).

In total, 7646 children presented to the health facilities in the BHDSS and met the surveillance criteria for clinician review (Fig 1). Sixty-one (0.8%) patients were not reviewed by a clinician and the residential status of 102 (1.3%) was undetermined. Of 6310 patients with suspected pneumonia, meningitis, or septicemia, 2358 (37%) were treated as outpatients and 3952 were admitted. Of those admitted, 155 (3.9%) died as inpatients. Twenty-four (0.6%) patients without an outcome measure and 38 (1.0%) with inadequate records were excluded with 3735 of 3797 eligible admissions (98%) remaining in the final analysis. Of those included in the analysis, 1602 (43%) were screened at peripheral primary care facilities outside Basse.

**Fig 2:**
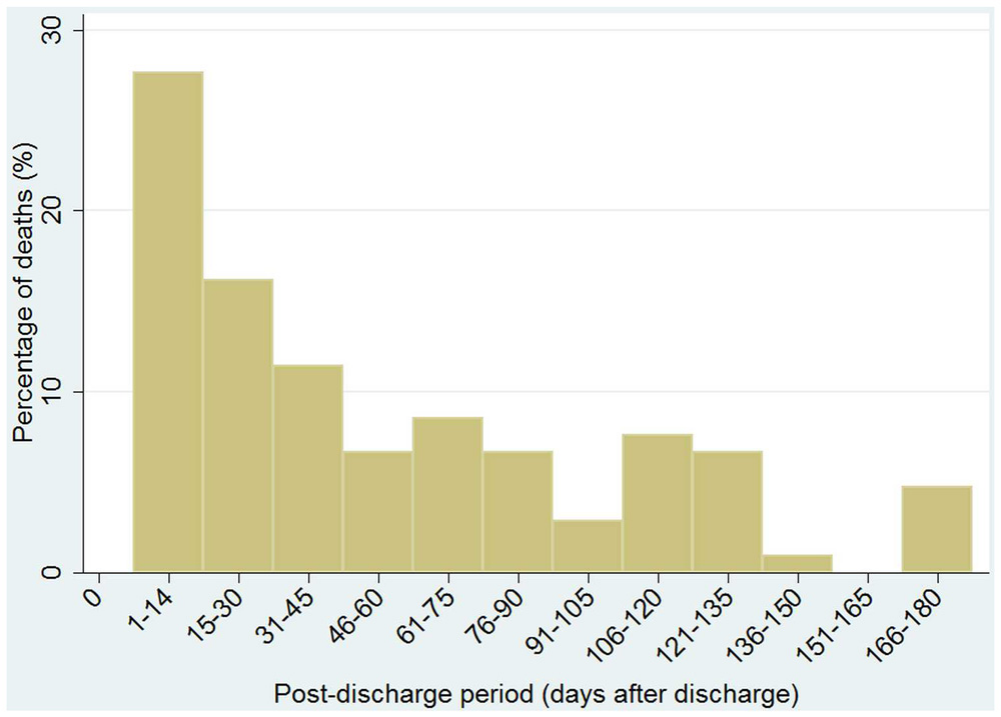
Temporal distribution of 105 deaths during 180 day follow-up after discharge from Basse Health Centre. Fifty-five patients exited alive from the Basse HDSS during 180 day follow-up.

During the 180 day follow-up period, there were 105 deaths among the 3735 (2.8%) admissions. Fifty-five patients exited alive from the BHDSS during 180 day follow-up, 22 in the first 90 days and 33 in the second 90 days. In the 2–11 month age group 47 of 1605 (2.9%) children died; in the 12–23 month age group 39 of 1127 (3.5%) died and there were 19 deaths among 1003 (1.9%) children aged 24–59 months. The crude mortality rate was 28.5 deaths/1000 discharges/180 days of follow-up. The mortality rate was 29.8 deaths/1000/180 days for children 2–11 months of age and 27.6 for children aged 12–59 months. The mortality rates in the background BHDSS populations aged 2–11 and 12–59 months during the period of the study were 11 and 5 deaths/1000/180 days respectively. Fifty-eight deaths (55%) occurred within 45 days of discharge and 81 (77%) occurred in the first 90 days (Fig 2).

On univariate analysis, children who died were more likely than children who survived to present in Basse than peripheral clinics, have a longer duration of illness, have no cough or difficulty breathing, have prostration, diarrhea, lower temperature, lower heart rate, lower respiratory rate, lower oxygen saturation, lower hemoglobin concentration, lower values of nutritional indices, lethargy, an absence of chest wall indrawing, nasal flaring, crackles, and wheeze, but have neck stiffness, a longer duration of admission, a standardised diagnosis of septicemia rather than pneumonia, multiple clinical diagnoses, bacteremia, be transferred from Basse, be not recovering on discharge and be discharged against medical advice (S3 Table).

**Fig 3:**
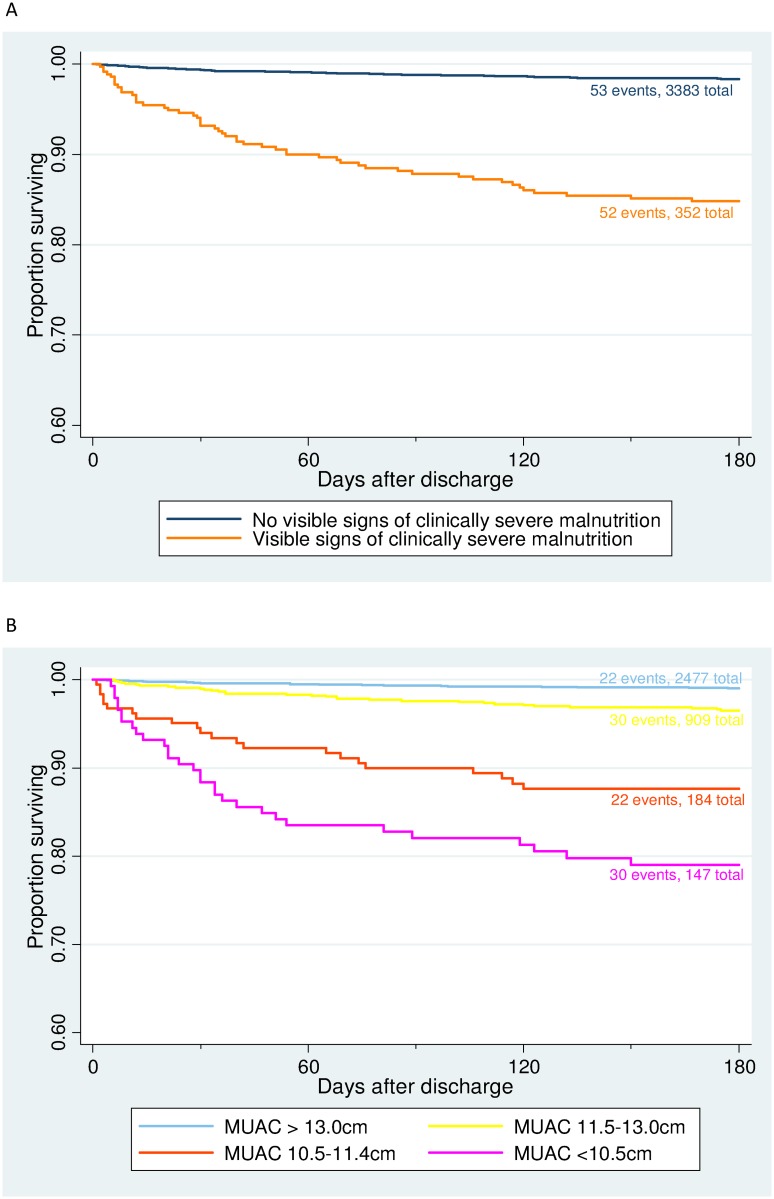
Kaplan-Meier survival estimates for 105 deaths during 180 day follow-up after discharge of 3735 children from Basse Health Centre, by categories of (A) clinically severe malnutrition and (B) mid-upper arm circumference. Clinically severe malnutrition was defined as the presence of visible wasting of the buttocks, characteristic skin or hair changes, or bilateral pedal oedema. Fifty-five patients exited alive from the Basse HDSS during 180 day follow-up. Numbers of events refer to the number of post-discharge deaths in each category. Total numbers refer to numbers of children in each category at the beginning of follow-up. Abbreviations: MUAC, mid-upper arm circumference (measured in centimetres).

The risk of death was reduced by 2% with each increasing month of age (Table 1). Clinically severe malnutrition was present in 52 of 105 (50%) children who died and 300 of 3630 (8.3%) who survived. The increased risk of death associated with CSM was greatest in the first 2 months after discharge but remained during months 2 to 6 of follow-up (Fig 3).

**Table 1:** Multi-variable Cox regression model for the hazard of post-discharge mortality associated with different clinical syndromes.

Risk factor	Survived or exited during180 day follow-up(N = 3630)	Died during 180day follow-up(N = 105)	Multivariate Coxhazard ratio (95% CI)	p-value
**Age in months** ^a^	18.0 (13.4)	15.7 (11.0)	0.98 (0.96 to 0.996)	0.02
**Sepsis with CSM** ^b^	134 (3.7%)	33 (31.4%)	18.4 (11.3 to 30.0)	<0.001
**Meningitis with CSM** ^b^	16 (0.4%)	3 (2.9%)^d^	13.7 (4.2 to 44.7)	<0.001
**Pneumonia with CSM** ^b^	150 (4.1%)	16 (15.2%)	8.1 (4.4 to 14.8)	<0.001
**Meningitis without CSM** ^b^	335 (9.2%)	9 (8.6%)	2.6 (1.2 to 5.5)	0.01
**Sepsis without CSM** ^b^	468 (12.9%)	12 (11.4%)	2.2 (1.1 to 4.3)	0.02
**Pneumonia without CSM** ^b^	2,527 (69.6%)	32 (30.5%)	1.00	-

^a^ Mean (Standard Deviation).

^b^ (CSM) Clinically severe malnutrition defined as visible wasting of the buttocks, characteristic skin or hair changes, or bilateral pedal oedema.

The multivariable model using clinical syndrome on admission showed that children with suspected pneumonia without CSM had the lowest risk of post-discharge mortality (Table 1). This risk was increased approximately two fold in children with suspected meningitis or septicaemia without CSM. The risk of post-discharge mortality was increased substantially in children with CSM. Compared to children with the clinical syndrome of pneumonia without CSM, those with pneumonia and CSM had 8 times (95% CI 4 to 15) the risk of death, those with suspected sepsis and CSM had 18 times (95% CI 11 to 30) the risk of death and those with suspected meningitis and CSM had 14 times (95% CI 4 to 45) the risk of death.

The multivariable model using demographic, clinical, and circumstantial factors showed that the risk of post-discharge mortality increased in a dose-dependent fashion with falling values of the MUAC on admission (Table 2). Compared to children with MUAC >13.0 cm, the risk of death increased 7 times with MUAC 11.5–13.0 cm, 24 times with MUAC 10.5–11.4 cm, and 44 times with MUAC <10.5 cm. The other factors which were associated with death in the risk factor model were: discharge in the dry season (HR 2.0, 95% CI 1.2 to 3.3), neck stiffness (HR 10, 95% CI 3 to 35), non-medical discharge (HR 4.7, 95% CI 2 to 11), while the risk of death was 29% (42 to 13%) lower for one unit increase in degrees Celsius of axillary temperature, 4% (1 to 7%) lower for one percent increase in oxygen saturation, and 18% (9% to 27%) lower for one unit increase in hemoglobin concentration. Age was not associated with the risk of death in this model. Mid-upper arm circumference ≤13.0 cm was present in 82 of 105 (78%) children who died and 1158 of 3630 (32%) children who survived. We repeated this model using categories of weight-for-height z-score instead of MUAC and found similar associations for the other risk factors but the magnitude of association with post-discharge mortality was less for z-scores (z-score < -3, HR 6.7, 95% CI 2.9 to 19.6; z-score ≤ -2 and ≥ -3, HR 2.7, 95% CI 1.3 to 5.8) than MUAC (S4 Table).

**Table 2:** Multi-variable Cox regression model for the hazard of post-discharge mortality associated with clinical characteristics, anthropometry using mid-upper arm circumference.

Risk factor	Survived or exitedduring 180 dayfollow-up (N = 3630)	Died during 180day follow-up(N = 105)	Multivariate Coxhazard ratio (95% CI)	p-value
**Age in months** ^a^	18.0 (13.4)	15.7 (11.0)	1.00 (0.98 to1.03)	0.13
**Dry season** ^b^	1889 (52%)	60 (57%)	1.96 (1.16 to 3.32)	0.01
**Neck stiffness**	30 (1%)	5 (5%)	10.4 (3.1 to 34.8)	<0.001
**Non-medical discharge**	47 (1%)	7 (7%)	4.68 (2.01 to 10.85)	<0.001
**Axillary temperature (** ^**o**^ **C)** ^a^	38.2 (1.4)	37.8 (1.2)	0.71 (0.58 to 0.87)	<0.001
**Oxygen saturation (%)** ^a^	94.6 (4.7)	92.5 (8.0)	0.96 (0.93 to 0.99)	0.01
**Haemoglobin concentration (g/dL)** ^a^	9.4 (2.0)	8.6 (2.4)	0.82 (0.73 to 0.91)	<0.001
**Mid-upper arm circumference**				
>13.0 cm	2,455 (68%)	22 (21%)	1.00	
11.5–13.0 cm	879 (24%)	30 (29%)	7.19 (3.04 to 17.01)	<0.001
10.5–11.4 cm	162 (4%)	22 (21%)	24.2 (9.4 to 61.9)	<0.001
<10.5 cm	117 (3%)	30 (29%)	43.7 (17.7 to 108)	<0.001

^a^ Mean (Standard Deviation).

^b^ Dry season (Dec-Jun). Note: Due to exclusion of participants with missing values 2013/3735 participants contributed to the final model.

Considering the prognostic value of CSM compared to MUAC ≤13.0 cm for post-discharge mortality, the specificity of the two indices was 92% versus 68%, while the sensitivity was 50% versus 78%. Exploring the utility of different measurements to define groups for potential interventions, categorisation of patients by MUAC ≤13.0 cm would include 33% of discharges and 77% of post-discharge deaths while MUAC <11.5 cm would include 9% of discharges and 50% of deaths. Categorising patients by CSM would include 9% of discharges and 50% of deaths while also including the clinical syndromes of meningitis and sepsis would include 31% of discharges and 70% of deaths. The number of patients that would be necessary to follow-up with a hypothetical 100% effective intervention in order to potentially prevent one death was 15 for MUAC <13.0 cm, 6 for MUAC <11.5 cm, 19 for weight-for-height *z*-score < -2, and 12 for weight-for-height *z*-score < -3.

## Discussion

To our knowledge, this is the first study of post-discharge mortality in a primary and secondary care setting in a developing country. The proportions of children 2–59 months of age admitted to a rural health centre with suspected pneumonia, sepsis, or meningitis, who died as inpatients (3.9%) or during post-discharge (2.7%) follow-up were similar. Post-discharge mortality in rural Gambia was five times higher than the background community rate. The majority of deaths occurred within 90 days of discharge. Children with signs of CSM on admission had an over 10-fold increase in the risk of post-discharge mortality. Post-discharge mortality was intimately related to increasing degrees of malnutrition as measured by MUAC, with a measurement of less than 10.5 cm associated with a 44-fold increase in the risk of mortality.

Our data align with previous studies of post-discharge mortality, demonstrating higher mortality than in background populations [9,10,12] and that mortality is highest soon after discharge [9, 10, 12, 17, 18]. Variability in the absolute rate of post-discharge mortality between our study and others may relate to our setting of primary and secondary care as well as differences in health care seeking, quality of care and duration of follow-up; 2.8% of children died in rural Gambia (6 months follow-up), whereas during 12 months of follow-up 4.5% died in coastal Kenya [12] and 6.1% in Guinea Bissau [10]. The setting of our study in primary and secondary care provides data representative of most admissions to health facilities in sub-Saharan Africa [19], thus confirming that post-discharge mortality comprises a major component of child mortality, unrelated to admissions in hospitals and referral centres.

In our clinical syndrome model, suspected diagnoses of sepsis or meningitis were associated with increased mortality following discharge, but risk was almost an order of magnitude greater when CSM was also present. Our risk factor model found that children with MUAC <11.5 cm on admission, indicative of severe malnutrition, had an extremely high risk of post-discharge death; 25 times greater than children with a normal MUAC. Importantly, a MUAC of 11.5–13.0 cm, indicative of moderate malnutrition, was also associated with a 7-fold increase in mortality. A number of studies have identified severe malnutrition as a risk factor for post-discharge mortality [9–12, 14, 15, 17, 18]. However, ours is the first reported study to identify an increased risk of mortality in relation to moderate malnutrition. Furthermore, we found that using MUAC as the anthropometric measure was more sensitive in categorising children at risk than weight-for-height z-scores. This is an important finding given the greater prevalence of moderate compared to severe malnutrition. The potential impact of an intervention would be greater if it included children with moderate as well as severe malnutrition.

The association between age and post-discharge mortality evident in our clinical syndrome model has also been shown by others [9–12], with older children generally having better outcomes. In contrast, age was not associated with post-discharge mortality in our risk factor model. The lack of association with age was due to mortality being greater in the 12–23 month (3.5%) than the 2–11 month age group (2.9%) which reflects the greater prevalence of malnutrition (weight-for-height z-score ≤ -2) in the older (38%, 411/1078) compared to younger (25%, 392/1564) age group. Although a study in Kenya found no relationship between post-discharge mortality and season [12], post-discharge mortality in Gambia was greater in the dry season, possibly related to a greater prevalence of invasive pneumococcal infections and rotavirus infection at this time [20, 21]. A recent systematic review of mortality in relation to seasonality concluded that deaths are more associated with humid and rainy seasons [22]. The reason for the apparent discrepancy between this review and our data may relate to differential risk for the major seasonal diseases in the specific post-discharge time period. Although assessed in previous research [12], we showed an association between neck stiffness on admission and post-discharge mortality, indicating some post-discharge deaths may be due to recrudescence of meningitis, and an increased risk in children with a low admission temperature. Children unable to mount a strong febrile response may be malnourished, immunosuppressed or moribund. Our findings that post-discharge mortality was associated with anaemia and hypoxia have not been consistent in other studies [10–12, 15]. If a discharge was noted to be against medical advice then there was an increased risk of post-discharge mortality. Findings from other studies in this regard are mixed [10, 18].

Moisi and colleagues reported that focussing on one or more characteristics significantly associated with post-discharge mortality (including weight-for-age z-score) would target 33% of discharged patients, include 47% of post-discharge deaths, and that the number of patients followed to identify one death would be 16 or less [12]. Our findings are similar to those from Kenya but in our setting a more simple approach may identify a greater proportion of post-discharge deaths. Categorising patients only by MUAC <13.0 cm would target 33% of discharges and 77% of post-discharge deaths whereas using MUAC <11.5 cm or CSM with or without clinical syndromes would target 9% to 31% of discharges identifying a maximum 70% of deaths. The use of MUAC is attractive as it is more simple to measure than height and weight and it is a well-accepted correlate of moderate (<13.0 and ≥11.5cm) and severe (<13.0cm) acute malnutrition [23, 24].

The strengths of our study are that all patients in the six facilities in the study area were screened for inclusion with clinical and follow-up information collected prospectively with little loss to follow-up. A limitation of our study is that we did not include the HIV status of patients. However, the low prevalence of HIV among antenatal women in Basse, around 1% [25], suggests that HIV will have had only a minor influence on post-discharge survival. The exclusion of participants from the model based on risk factors, primarily due to missing haemoglobin values among patients enrolled before 2011, may have under-estimated the association as measurements of haemoglobin would be biased towards lower values. Our study excluded around 22% of patients who were admitted in the BHDSS. Thus, even though we followed the majority of discharged patients our data do not provide information about children with simple diarrhoea and dehydration or those with malaria without neurologic complications (patients with malaria and neurologic complications were categorised as suspected meningitis).

Our study shows that post-discharge mortality is substantial in primary and secondary health care in West Africa. Children with CSM had poorer outcomes post-discharge, particularly if they had a more severe provisional diagnosis on admission. Furthermore, we have shown that moderate malnutrition is a significant risk factor for post-discharge death and that measurement of MUAC provides the most sensitive approach to detect high-risk children. This relatively neglected group of children deserves more attention. Preventive interventions should be tested and might include nutritional rehabilitation in the community, prophylactic chemotherapy, or improved systems for the follow-up of at risk children.

## Supporting Information

S1 Fig Map of Africa showing The Gambia in West Africa with the study area in the east of the country in Upper River Region. The Basse Health and Demographic Surveillance System (BHDSS) is highlighted in pink and Basse town is indicated. The URR is bisected into north and south banks by The Gambia River.
(TIFF)

S1 Panel Guidelines for investigation of suspected pneumonia, meningitis, or septicaemia (DOCX)

S1 Table Criteria for clinician review for assessment of suspected pneumonia, meningitis, or septicaemia. (DOCX)

S2 Table Clinical definitions for suspected pneumonia, meningitis, or septicaemia. (DOCX)

S3 Table Univariate Cox survival analysis of the hazard of post-discharge mortality association with demographic and clinical patient characteristics. ^a^ SD = standard deviation. ^b^ Numbers do not sum to the total number of participants due to missing values. ^c^ Complete PCV (pneumococcal conjugate vaccine) vaccination defined as receipt of three doses or if less than three doses given doses given within 1 month of the scheduled age. ^d^ Days unwell is the reported length of illness at presentation. ^e^ Prostration defined as loss of the ability to sit, drink, or breastfeed. ^f^ Numbers in square brackets refers to number of participants with valid information for the variable.
(DOCX)

S4 Table Multi-variable Cox regression model for the hazard of post-discharge mortality associated with clinical characteristics, anthropometry using weight-for-height. ^a^ Mean (Standard Deviation). ^b^ Dry season (Dec-Jun). Note: Due to exclusion of participants with missing values 1954 participants contributed to the model.
(DOCX)
