# Supplementary figures and images for: Child Mortality after Discharge from a Health Facility following Suspected Pneumonia, Meningitis or Septicaemia in Rural Gambia: A Cohort Study
Source: PLoS One. 2015 Sep 9;10(9):e0137095. doi: 10.1371/journal.pone.0137095 (PMC4564213; doi:10.1371/journal.pone.0137095)

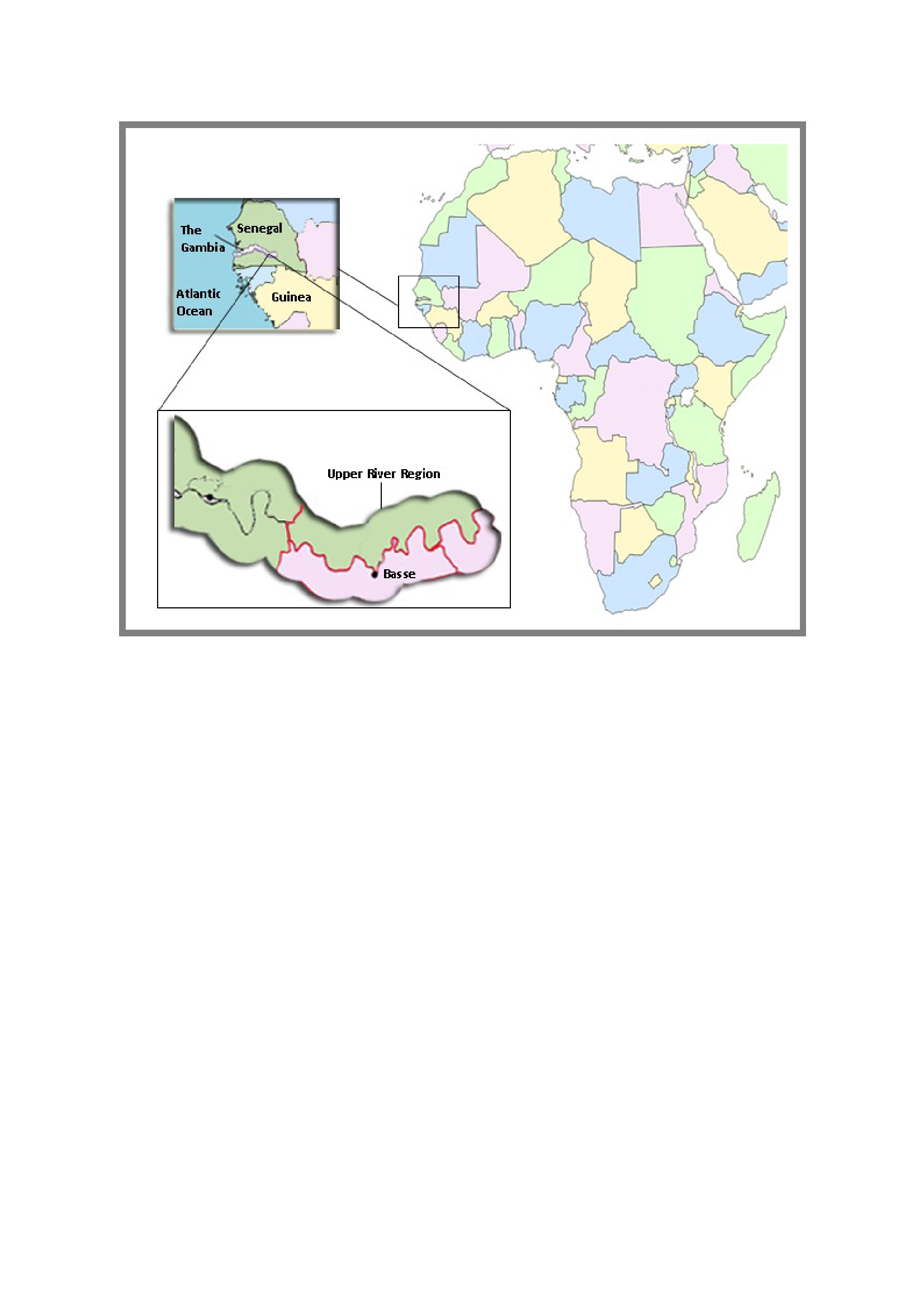

Supplement: S1 Fig — The Basse Health and Demographic Surveillance System (BHDSS) is highlighted in pink and Basse town is indicated. The URR is bisected into north and south banks by The Gambia River. (TIFF) [file pone.0137095.s001.tiff]
