# Supplementary material for: Child Mortality after Discharge from a Health Facility following Suspected Pneumonia, Meningitis or Septicaemia in Rural Gambia: A Cohort Study
Source: PLoS One. 2015 Sep 9;10(9):e0137095. doi: 10.1371/journal.pone.0137095 (PMC4564213; doi:10.1371/journal.pone.0137095)
Supplement: S1 Panel — (DOCX) [file pone.0137095.s002.docx]

SPanel 1. Guidelines for investigation of suspected pneumonia, meningitis, or septicaemia.

The following guide applies to the investigation of children and adults diagnosed with suspected meningitis, septicaemia, and meningitis, according to the above criteria, as well as children 0-59 months of age enrolled in non-referral surveillance:

Patients with suspected pneumococcal disease are to have blood culture.

Non-referral surveillance patients being admitted are to have blood culture.

Patients with suspected pneumococcal disease are to have,

A rapid diagnostic test for malaria (from january – july the rdt is only done if the surveillance number ends in ‘0’).

Serum collection for antibiotic activity detection if the surveillance number ends in ‘0’ or ‘5’ and the patient is enrolled in basse.

Patients with suspected meningitis are to have lumbar puncture.

Patients with suspected pneumonia are to have chest x-ray.

Chest x-ray should also be considered in patients with meningitis or septicaemia if the clinician’s impression is of co-existing pneumonia or if it is judged that a chest x-ray will assist in management.

Lung aspirate should be considered for a patient if peripheral consolidation has been demonstrated, preferably by x-ray.

Other investigations including pleural tap and joint aspirate may be considered according to the clinical indication.
