# Supplementary material for: Child Mortality after Discharge from a Health Facility following Suspected Pneumonia, Meningitis or Septicaemia in Rural Gambia: A Cohort Study
Source: PLoS One. 2015 Sep 9;10(9):e0137095. doi: 10.1371/journal.pone.0137095 (PMC4564213; doi:10.1371/journal.pone.0137095)
Supplement: S1 Table — (DOCX) [file pone.0137095.s003.docx]

STable 1. Criteria for clinician review for assessment of suspected pneumonia, meningitis, or septicaemia

| **Age ≥2 months and <5 years** | **Age ≥5 years** |
| --- | --- |
| To be referred for further assessment if one or more of the following are present for 14 days or less:   1. History of cough^1^ or difficulty breathing, AND raised respiratory rate for age^2^ 2. Axillary temperature^3^ of at least 38°C, or less than 36°C in a patient admitted or being admitted 3. History of convulsion 4. Impaired consciousness^4^ 5. Bulging fontanelle 6. Stiff neck 7. Prostration^5^ 8. Lower chest wall indrawing, nasal flaring, or grunting 9. Oxygen saturation less than 92% 10. Weight below -3 z-score for age 11. Local musculoskeletal swelling or tenderness 12. Irrespective of residential location, any child with suspected meningitis | to be referred for further assessment if one or more of the following are present for 14 days or less:  History of cough and difficulty breathing  History of cough and pleuritic chest pain  History of cough and supraclavicular/sternal recession or nasal flaring  History of productive cough and fever  History of rigors  History of seizure  Impaired consciousness^4^  Altered mental state  Axillary temperature^3^ of at least 38°c or less than 36°c in a patient admitted or being admitted  Photophobia  Neck stiffness   1. Local musculoskeletal swelling or tenderness 2. Irrespective of residential location, any patient with suspected meningitis |

**Notes**

^1^Cough includes a dry cough, sputum production, or haemoptysis.

^2^Raised respiratory rate for age is defined as greater than 50 breaths per minute for children at least 2 months but less than 12 months, and as greater than 40 breaths per minute for children at least 12 months but less than 60 months.

^3^Axillary temperature: This criteria initially included outpatients and was then modified to refer only to inpatients at Basse Health Centre.

^4^Impaired consciousness is defined as V, P, or U on the AVPU score, where A is if the patient is alert, V if responsive to verbal stimulus, P if responsive to pain stimulus, and U if unresponsive.

^5^Prostration is defined as inability to drink or breast feed, or to remain sitting in a child otherwise able to sit.
