# Supplementary material for: Child Mortality after Discharge from a Health Facility following Suspected Pneumonia, Meningitis or Septicaemia in Rural Gambia: A Cohort Study
Source: PLoS One. 2015 Sep 9;10(9):e0137095. doi: 10.1371/journal.pone.0137095 (PMC4564213; doi:10.1371/journal.pone.0137095)
Supplement: S2 Table — (DOCX) [file pone.0137095.s004.docx]

STable 2. Clinical definitions for suspected pneumonia, meningitis, or septicaemia.

|  | **Age ≥2 months and <5 years** | **Age ≥5 years** |
| --- | --- | --- |
| **Suspected pneumonia** | Suspected pneumonia is defined if there is a history of cough or difficulty breathing of less than 14 days’ duration, accompanied by one or more of:  1. Raised respiratory rate for age^1^  2. Lower chest wall indrawing, nasal flaring or grunting  3. Oxygen saturation less than 92%  4. Focal chest signs (dull percussion note, coarse crackles, bronchial breathing) | Suspected pneumonia will be defined according to clinical judgement and is to be considered in patients presenting with an illness of 14 days’ duration or less, if two or more of the following are present::  1. Cough  2. Haemoptysis  3. Pleuritic chest pain  4. Breathlessness  5. Axillary temperature ≥38°C |
| **Suspected meningitis** | Suspected meningitis will be defined according to clinical judgement and is to be considered if any of the following are present:  1. Neck stiffness  2. Impaired consciousness^2^  3. Prostration^3^  4. History of convulsion  5. Bulging fontanelle | Suspected meningitis will be defined according to clinical judgement and is to be considered if two or more of the following are present:  1. Axillary temperature ≥38°C  2. Meningism (neck stiffness and/or photophobia)  3. Altered mental state (Glasgow Coma Score <14) |
| **Suspected septicaemia** | Suspected septicaemia will be defined as one or more of:  1. Clinician diagnosis of focal sepsis (including but not limited to: septic arthritis, osteomyelitis, endocarditis, peritonitis, liver abscess, soft tissue abscess, cellulitis)  2. Axillary temperature is <36°C or ≥38°C and no obvious cause of fever  3. History of rigors  4. For a patient admitted, or being admitted, the clinical impression is of severe malnutrition^4^. | Suspected septicaemia will be defined as one or more of:  1. Clinician diagnosis of focal sepsis (including but not limited to: septic arthritis, osteomyelitis, endocarditis, peritonitis, liver abscess, soft tissue abscess, cellulitis)  2. Axillary temperature is <36°C or ≥38°C and no obvious cause of fever  3. History of rigors |

**Notes**

Raised respiratory rate for age is defined as greater than 50 breaths per minute for children at least 2 months but less than 12 months, and as greater than 40 breaths per minute for children at least 12 months but less than 60 months.

^2^Impaired consciousness is defined as V, P, or U on the AVPU score, where A is if the patient is alert, V if responsive to verbal stimulus, P if responsive to pain stimulus, and U if unresponsive.

^3^Prostration is defined as inability to drink or breast feed, or to remain in a seated position in a child otherwise able to do so.

^4^Severe malnutrition is defined as the presence of visible wasting of the buttocks, characteristic skin or hair changes, or bilateral pedal oedema.
