# Supplementary material for: Child Mortality after Discharge from a Health Facility following Suspected Pneumonia, Meningitis or Septicaemia in Rural Gambia: A Cohort Study
Source: PLoS One. 2015 Sep 9;10(9):e0137095. doi: 10.1371/journal.pone.0137095 (PMC4564213; doi:10.1371/journal.pone.0137095)
Supplement: S3 Table — a SD = standard deviation. b Numbers do not sum to the total number of participants due to missing values. c Complete PCV (pneumococcal conjugate vaccine) vaccination defined as receipt of three doses or if less than three doses given doses given within 1 month of the scheduled age. d Days unwell is the reported length of illness at presentation. e Prostration defined as loss of the ability to sit, drink, or breastfeed. f Numbers in square brackets refers to number of participants with valid information for the variable. (DOCX) [file pone.0137095.s005.docx]

STable 3. Univariate Cox survival analysis of the hazard of post-discharge mortality association with demographic and clinical patient characteristics.

| Risk factor | Survived or exited during 180 day follow-up  (N=3630) | Died during 180 day follow-up  (N=105) | Univariate Cox  hazard ratio  [95% CI] | p-value |
| --- | --- | --- | --- | --- |
| Age in months Mean (SD^a^) | 18.0 (13.4) | 15.7 (11.0) | 0.99 [0.97, 1.00] | 0.09 |
| Age group 2-23 months n (%) | 2,646 (73%) | 86 (82%) | 1 |  |
| 24-59 months n (%) | 984 (27%) | 19 (18%) | 0.61 [0.37, 0.996] | 0.048 |
| Sex^b^ Female n (%) | 1,565 (43%) | 49 (47%) | 1.17 [0.79, 1.71] | 0.43 |
| Ethnicity^b^ Fula n (%) | 949 (26%) | 23 (22%) | 1 | - |
| Mandinka n (%) | 929 (26%) | 34 (32%) | 1.52 [0.89, 2.58] | 0.12 |
| Sarahule n (%) | 1,664 (46%) | 45 (43%) | 1.12 [0.68, 1.85] | 0.66 |
| Wolof & Other n (%) | 73 (2%) | 2 (2%) | 1.11 [0.26, 4.71] | 0.89 |
| Screened at outlying clinic Yes n (%) | 1,569 (43%) | 33 (31%) | 0.61 [0.41, 0.93] | 0.02 |
| PCV completeness^c^ Complete n (%) | 1,563 (43%) | 40 (38%) | 1 | - |
| Partial n (%) | 473 (13%) | 17 (16%) | 1.33 [0.76, 2.35] | 0.32 |
| None n (%) | 1,594 (44%) | 48 (46%) | 1.08 [0.71, 1.65] | 0.71 |
| Days unwell^d^ 0-3 days n (%) | 2,547 (70%) | 45 (43%) | 1 | - |
| 4-7 days n (%) | 958 (26%) | 44 (42%) | 2.54 [1.68, 3.85] | <0.001 |
| >7 days n (%) | 124 (3%) | 15 (14%) | 6.43 [3.58, 11.53] | <0.001 |
| History of cough Yes n (%) | 3,356 (92%) | 90 (86%) | 0.48 [0.28, 0.83] | 0.01 |
| History of difficulty in breathing Yes n (%) | 2,795 (77%) | 66 (63%) | 0.51 [0.34, 0.75] | <0.001 |
| History of irritability Yes n (%) | 778 (21%) | 22 (21%) | 1.01 [0.63, 1.62] | 0.96 |
| History of prostration^e^ Yes n (%) | 276 (8%) | 16 (15%) | 2.17 [1.27, 3.7] | <0.001 |
| History of convulsion Yes n (%) | 313 (9%) | 9 (9%) | 0.98 [0.49, 1.93] | 0.95 |
| History of fever Yes n (%) | 3,500 (96%) | 101 (96%) | 0.94 [0.35, 2.55] | 0.90 |
| History of diarrhoea Yes n (%) | 827 (23%) | 36 (34%) | 1.72 [1.15, 2.58] | 0.01 |
| History of vomiting Yes n (%) | 862 (24%) | 24 (23%) | 0.93 [0.59, 1.47] | 0.75 |
| Antibiotics taken in previous week? Yes n (%) | 336 (9%) | 13 (12%) | 1.41 [0.78, 2.55] | 0.25 |
| Unknown n (%) | 710 (20%) | 21 (20%) | 1.08 [0.66, 1.75] | 0.77 |
| Axillary temperature, ^o^C | [n=3630] **^f^** | [n=105] |  |  |
| Mean (SD^a^) | 38.2 (1.4) | 37.8 (1.2) | 0.72 [0.61, 0.84] | <0.001 |
| Pulse rate, beats/min | [n=3624]^f^ | [n=105] |  |  |
| Mean (SD^a^) | 156.1 (22.0) | 148.0 (29.6) | 0.98 [0.98, 0.99] | <0.001 |
| Respiratory rate, breaths/min | [n=3629]^f^ | [n=104] |  |  |
| Mean (SD^a^) | 59.4 (17.4) | 54.8 (21.4) | 0.98 [0.97, 0.99] | <0.001 |
| Oxygen saturation, % | [n=2736]^f^ | [n=72] |  |  |
| Mean (SD^a^) | 94.6 (4.7) | 92.5 (8.0) | 0.95 [0.93, 0.98] | <0.001 |
| Clinically severe malnutrition Yes n (%) | 300 (8%) | 52 (50%) | 9.89 [6.75, 14.5] | <0.001 |
| Haemoglobin concentration, g/dL | [n=2291]^f^ | [n=76] |  |  |
| Mean (SD^a^) | 9.4 (2.0) | 8.6 (2.4) | 0.85 [0.77, 0.94] | <0.001 |
| Mid upper arm circumference >13.0 cm n (%) | 2,455 (68%) | 22 (21%) | 1 |  |
| 11.5-13.0 cm n (%) | 879 (24%) | 30 (29%) | 3.71 [2.14, 6.43] | <0.001 |
| 10.5-11.4 cm n (%) | 162 (4%) | 22 (21%) | 14.13 [7.83, 25.52] | <0.001 |
| <10.5 cm n (%) | 117 (3%) | 30 (29%) | 25.38 [14.64, 44.00] | <0.001 |
| Height-for-age z-score | [n=3526]^f^ | [n=96] |  |  |
| Mean (SD^a^) | -1.2 (1.5) | -2.5 (1.9) | 0.57 [0.51, 0.65] | <0.001 |
| Weight-for-age z-score | [n=3568]^f^ | [n=92] |  |  |
| Mean (SD^a^) | -1.6 (1.4) | -3.4 (1.6) | 0.48 [0.42, 0.55] | <0.001 |
| Weight-for-height z-score | [n=3511]^f^ | [n=87] |  |  |
| Mean (SD^a^) | -1.3 (1.4) | -2.6 (1.5) | 0.51 [0.44, 0.60] | <0.001 |
| Lethargy Yes n (%) | 877 (24%) | 49 (47%) | 2.62 [1.78, 3.84] | <0.001 |
| Grunting Yes n (%) | 508 (14%) | 11 (10%) | 0.7 [0.37, 1.3] | 0.26 |
| Lower chest wall in-drawing Yes n (%) | 2,300 (63%) | 54 (51%) | 0.58 [0.4, 0.85] | 0.01 |
| Nasal flaring Yes n (%) | 1,918 (53%) | 41 (39%) | 0.56 [0.38, 0.83] | <0.001 |
| Crackles Yes n (%) | 1,858 (51%) | 40 (38%) | 0.6 [0.41, 0.89] | 0.01 |
| Wheeze Yes n (%) | 910 (25%) | 13 (12%) | 0.42 [0.23, 0.74] | <0.001 |
| Bronchial breathing Yes n (%) | 498 (14%) | 12 (11%) | 0.87 [0.48, 1.58] | 0.64 |
| Dull percussion note Yes n (%) | 437 (12%) | 16 (15%) | 1.25 [0.73, 2.13] | 0.41 |
| Bulging fontanelle Yes n (%) | 38 (1%) | 1 (1%) | 0.95 [0.13, 6.82] | 0.96 |
| Neck stiffness Yes n (%) | 30 (1%) | 5 (5%) | 6.16 [2.51, 15.13] | <0.001 |
| Length of admission, in days | [n= 3557]^f^ | [n=103] |  |  |
| Mean (SD^a^) | 3.7 (2.5) | 6.2 (4.4) | 1.19 [1.14, 1.23] | <0.001 |
| Number of admissions during follow-up | [n=3630]^f^ | [n=105] |  |  |
| Mean (SD^a^) | 1.4 (0.8) | 1.3 (0.5) | 0.77 [0.56,1.06] | 0.11 |
| Standardised surveillance diagnosis |  |  |  |  |
| Pneumonia n (%) | 2,677 (74%) | 48 (46%) | 1 |  |
| Meningitis n (%) | 233 (6%) | 5 (5%) | 1.19 [0.47, 2.98] | 0.71 |
| Septicaemia n (%) | 298 (8%) | 28 (27%) | 5.12 [3.21, 8.16] | <0.001 |
| Other focal sepsis n (%) | 32 (1%) | 2 (2%) ^b^ | 3.68 [0.89, 15.14] | 0.07 |
| Pneumonia & meningitis n (%) | 65 (2%) | 4 (4%)^b^ | 3.4 [1.23, 9.44] | 0.02 |
| Pneumonia & septicaemia n (%) | 272 (7%) | 15 (14%) | 3.27 [1.83, 5.84] | <0.001 |
| Meningitis & septicaemia n (%) | 31 (1%) | 2 (2%)^b^ | 3.5 [0.85, 14.42] | 0.08 |
| Pneumonia & meningitis & septicaemia n (%) | 22 (1%) | 1 (1%)^b^ | 2.58 [0.36, 18.73] | 0.35 |
| Number of diagnoses One n (%) | 2,208 (61%) | 46 (44%) | 1 |  |
| Two n (%) | 1,139 (31%) | 39 (37%) | 1.58 [1.03, 2.42] | 0.04 |
| Three n (%) | 235 (6%) | 17 (16%) | 3.19 [1.83, 5.57] | <0.001 |
| Four n (%) | 48 (1%) | 3 (3%)^b^ | 2.76 [0.86, 8.87] | 0.09 |
| Malaria test result Negative n (%) | 2,460 (68%) | 71 (68%) | 1 |  |
| Positive n (%) | 251 (7%) | 5 (5%) | 0.68 [0.27, 1.67] | 0.4 |
| Not done n (%) | 856 (24%) | 25 (24%) | 0.99 [0.62, 1.55] | 0.95 |
| Bacteraemia Yes n (%) | 284 (8%) | 17 (16%) | 2.28 [1.36, 3.83] | <0.001 |
| Type of separation Discharged n (%) | 3,589 (99%) | 97 (92%) | 1 |  |
| Transferred n (%) | 41 (1%) | 8 (8%) | 6.84 [3.32, 14.06] | <0.001 |
| Season of discharge Wet (Jul-Nov) n (%) | 1,741 (48%) | 45 (43%) | 1 |  |
| Dry (Dec-Jun) n (%) | 1,889 (52%) | 60 (57%) | 1.34 [0.91, 1.97] | 0.14 |
| Recovering on discharge Yes n (%) | 3,516 (97%) | 88 (84%) | 0.18 [0.11, 0.3] | <0.001 |
| Non-medical discharge Yes n (%) | 47 (1%) | 7 (7%) | 5.12 [2.38, 11.03] | <0.0011 |
| ^a^ SD = standard deviation. ^b^ Numbers do not sum to the total number of participants due to missing values. ^c^ Complete PCV (pneumococcal conjugate vaccine) vaccination defined as receipt of three doses or if less than three doses given doses given within 1 month of the scheduled age. ^d^ Days unwell is the reported length of illness at presentation. ^e^ Prostration defined as loss of the ability to sit, drink, or breastfeed. ^f^ Numbers in square brackets refers to number of participants with valid information for the variable. | | | | |
