# Supplementary material for: Child Mortality after Discharge from a Health Facility following Suspected Pneumonia, Meningitis or Septicaemia in Rural Gambia: A Cohort Study
Source: PLoS One. 2015 Sep 9;10(9):e0137095. doi: 10.1371/journal.pone.0137095 (PMC4564213; doi:10.1371/journal.pone.0137095)
Supplement: S4 Table — a Mean (Standard Deviation). b Dry season (Dec-Jun). Note: Due to exclusion of participants with missing values 1954 participants contributed to the model. (DOCX) [file pone.0137095.s006.docx]

eTable 4. Multi-variable Cox regression model for the hazard of post-discharge mortality associated with clinical characteristics, anthropometry using weight-for-height

| Risk factor | Survived or exited during 180 day follow-up  (N=3630) | Died during 180 day follow-up  (N=105) | Multivariate Cox  hazard ratio [95% CI] | p-value |
| --- | --- | --- | --- | --- |
| Age in months^a^ | 18.0 (13.4) | 15.7 (11.0) | 0.97 [0.95,1.0] | 0.05 |
| Dry season^b^ | 1889 (52%) | 60 (57%) | 2.23 [1.26, 3.95] | 0.01 |
| Neck stiffness | 30 (1%) | 5 (5%) | 7.71 [2.34, 25.4] | <0.001 |
| Non-medical discharge | 47 (1%) | 7 (7%) | 7.49 [2.86, 19.6] | <0.001 |
| Axillary temperature (^o^C)^a^ | 38.2 (1.4) | 37.8 (1.2) | 0.71 [0.57, 0.88] | <0.001 |
| Oxygen saturation (per %)^a^ | 94.6 (4.7) | 92.5 (8.0) | 0.96 [0.93, 0.99] | 0.01 |
| Haemoglobin concentration (g/dL)^a^ | 9.4 (2.0) | 8.6 (2.4) | 0.80 [0.72, 0.90] | <0.001 |
| Weight-for-height |  |  |  |  |
| z-score ≥ -2 | 2,468/3511 (70%) | 29/87 (33%) | 1.00 |  |
| -2 ≤ z-score ≤ -3 | 626/3511 (18%) | 19/87 (22%) | 2.74 [1.30, 5.80] | 0.01 |
| z-score < -3 | 417/3511 (12%) | 39/87 (45%) | 6.73 [3.59, 12.6] | <0.001 |

^a^ Mean (Standard Deviation). ^b^ Dry season (Dec-Jun). Note: Due to exclusion of participants with missing values 1954 participants contributed to the model.
